# Profiles and Predictors of Family Functioning in Families of Children with Mowat–Wilson Syndrome: A Cross-Sectional Survey

**DOI:** 10.3390/healthcare14070931

**Published:** 2026-04-02

**Authors:** Nan Peng, Bo Zhou, Biyun Li, Qi Xu, Jianhong Wang, Lili Zhang, Weiheng Yan, Xia Qu, Lingya Liu, Bo Li, Qian Jiang, Sha Tao, Lin Wang

**Affiliations:** 1 Capital Institute of Pediatrics, Chinese Academy of Medical Sciences & Peking Union Medical College, Beijing 100020, China; b2023032008@pumc.edu.cn; 2 Department of Child Health Care, Capital Institute of Pediatrics, Capital Center for Children’s Health, Capital Medical University, Beijing 100020, China; bobo-0207@hotmail.com (B.Z.); lby9616@yeah.net (B.L.); cookie7826@mail.ccmu.edu.cn (Q.X.); child811117@163.com (J.W.); huaxiang1978@mail.ccmu.edu.cn (L.Z.); ywh_tmu@126.com (W.Y.); bestwhite@alumni.sjtu.edu.cn (X.Q.); 3 Department of Medical Genetics, Capital Institute of Pediatrics, Capital Center for Children’s Health, Capital Medical University, Beijing 100020, China; ivglly@163.com (L.L.); jiangq@mail.ccmu.edu.cn (Q.J.); 4 Precision Regenerative Medicine Research Centre, Medical Sciences Division, Macau University of Science and Technology, Macao 999078, China; bo.li@sickkids.ca; 5 State Key Laboratory of Cognitive Neuroscience and Learning, Beijing Normal University, Beijing 100875, China; 6 IDG/McGovern Institute for Brain Research, Beijing Normal University, Beijing 100875, China

**Keywords:** Mowat–Wilson syndrome, family assessment device, household income, caregiver burden

## Abstract

**Introduction**: Family functioning is a critical factor linked to child outcomes in neurodevelopmental disorders but remains unstudied in Mowat–Wilson syndrome (MWS), a rare condition with substantial caregiver burden. This study characterized family functioning in MWS and identified factors associated with family functioning. **Materials and Methods**: We employed a cross-sectional survey in a cohort of 42 patients with MWS. Family functioning was assessed using the Family Assessment Device (FAD). Clinical and behavioral characteristics included intellectual disability, adaptive functioning, autism spectrum disorder (ASD)-related behaviors, and CBCL-derived domains of social, emotional, and sleep-related concerns. **Results**: Among 42 children (mean age 4.9 years), severe/profound intellectual disability and adaptive deficits were common. Difficulties in family functioning were frequently reported. The highest proportions of scores above the published cut-off were observed in behavior control (90.5%) and affective involvement (76.2%). Higher monthly household income was independently associated with better general family functioning (β = −0.443, *p* = 0.008), whereas child age, intellectual disability severity, and adaptive impairment were not significantly associated with general functioning scores. Poorer general functioning was associated with higher scores on the CBCL-derived depressive symptom domain. **Conclusions**: Caregivers of children with MWS commonly reported difficulties in family functioning; behavior control was most affected, followed by affective involvement. Higher household income was independently associated with better general family functioning, suggesting that household income is an important socioeconomic correlate of family functioning.

## 1. Introduction

Mowat–Wilson syndrome (MWS) is a rare neurodevelopmental disorder resulting from heterozygous loss-of-function variants or deletions in the *ZEB2* gene (zinc finger E-box binding homeobox 2) [1], with an estimated incidence of 1/50,000–1/70,000 [2]. It is typically characterized by distinctive facial dysmorphism, moderate-to-severe intellectual disability (ID), global developmental delay (GDD), and multiple congenital anomalies. In addition to core neurodevelopmental impairments, affected children often require long-term management of multi-system comorbidities (epilepsy, feeding problems, sleep disturbances, and recurrent respiratory issues) [3]. This clinical profile often necessitates frequent multidisciplinary follow-up, long-term pharmacological and rehabilitative care, and substantial assistance with activities of daily living, thus resulting in a high and persistent caregiving burden.

Growing evidence underscores a dynamic and reciprocal relationship between neurodevelopmental conditions and family functioning. A longitudinal study in autism spectrum disorder (ASD) reported bidirectional associations between ASD characteristics and family functioning and that greater ASD trait severity predicted poorer family functioning [4]. A similar vicious cycle of bidirectional deterioration has also been observed in families of children with attention-deficit/hyperactivity disorder (ADHD), whereby child symptom severity and family functioning mutually exacerbate one another [5,6]. Moreover, the quality of family functioning is directly linked to the effectiveness of disease management. A meta-analysis demonstrated that higher adaptive family functioning (lower conflict and greater cohesion) is associated with significantly better treatment adherence among children with a range of chronic conditions [7]. In cerebral palsy, robust family functioning has been identified as the strongest protective factor against parental distress, with an impact that may even surpass certain forms of socioeconomic support [8]. Collectively, these findings highlight family functioning as a key construct for understanding family adaptation and as a potential target for interventions aimed at improving outcomes for affected children and their families.

Although the caregiving burden for children with MWS may be particularly high given the complexity and severity of their clinical presentation, no study to date has specifically examined family functioning in MWS. Findings from other pediatric conditions offer a useful conceptual framework for identifying candidate correlates of family functioning. Child-related clinical factors, particularly emotional and behavioral problems [9] and adaptive functioning [10], have consistently been linked to poorer family functioning and greater parental stress. Conversely, family resources, including parents’ social–ecological resilience [11] and social support [12], may mitigate stress and promote adaptive adjustment. However, it remains unclear how these factors operate within families of children with MWS. Accordingly, this study aimed to characterize family functioning in families of children with MWS using the Family Assessment Device (FAD), with primary emphasis on the general functioning subscale and exploratory description of the other six domains (problem solving, communication, roles, affective responsiveness, affective involvement, and behavior control). The study also examined factors associated with family functioning in this cohort. By describing overall family functioning and exploratory domain-level patterns, this study sought to provide preliminary evidence to inform future family-centered support strategies for families of children with MWS.

## 2. Materials and Methods

### 2.1. Patient Recruitment

We conducted a cross-sectional study. All patients included in this study were diagnosed with MWS at their respective local medical institutions using the same genetic testing approach and were subsequently seen for clinical care at the Capital Institute of Pediatrics between 2023 and 2025. Families were contacted and recruited by the investigators through online communication, and oral informed consent was obtained from caregivers prior to participation. During the study period, 46 families were contacted, and 42 patients were ultimately enrolled (response/enrollment rate: 91.3%).

Although the sample was recruited at a single center and should be considered a convenience sample, MWS is a rare disorder and, to the best of our knowledge, the enrolled patients account for a substantial proportion of the currently diagnosed MWS cases in China. Therefore, this sample may capture a relatively broad range of characteristics among currently identified Chinese patients with MWS while not necessarily representing all individuals with MWS.

Before the questionnaire link was distributed, verbal informed consent was obtained from the primary caregiver using a standardized telephone script. The script included confirmation of caregiver eligibility, an explanation of the study purpose and procedures, a description of potential risks and benefits, statements regarding the voluntary nature of participation and the right to withdraw at any time, and investigator contact information. It also included explicit verbal consent questions to confirm the caregiver’s understanding of the study and willingness to participate. The investigator documented the caregiver’s responses and verbal agreement before enrollment. To protect participant identity during online data collection, each participant was assigned a study identification number, and no names were entered into the questionnaire dataset. Data were stored on secure systems, and study findings were only reported in aggregate form to minimize the risk of identifying individual families. No medical record review was performed in this study.

### 2.2. Assessment of Neurodevelopment

Participants were recruited and administered standardized cognitive assessments to determine intelligence quotient (IQ) or developmental quotient (DQ) during routine follow-up visits at the Capital Institute of Pediatrics. All assessments were conducted in person in a dedicated, quiet testing room, using standardized materials and instructions according to the test manuals. The tests were administered by developmental pediatricians who had received formal training and were experienced in the use of the Gesell Developmental Schedules and the Wechsler Intelligence Scales. The examiners were blinded to the participants’ clinical information and study outcomes at the time of testing. Caregivers were allowed to stay with the child if needed but were instructed not to prompt or assist. Short breaks were offered to minimize fatigue and maintain engagement, and testing was rescheduled if the child was unwell or unable to complete the session. Raw scores were converted into age-standardized scores using the corresponding normative data.

Parental consent for cognitive testing was declined in 12 cases. Therefore, IQ/DQ assessment could not be performed for these participants. Finally, complete IQ/DQ data were obtained for 30 participants. Baseline characteristics of participants who consented versus declined were compared to assess potential selection bias. For children aged 0–6 years, the Gesell Developmental Schedules (GDS) were employed to evaluate DQ. The GDS are a widely recognized tool for assessing neurodevelopmental status in early childhood and serves as a standardized measure for identifying intellectual impairment within this age group [13]. For participants older than 6 years, IQ was assessed using age-appropriate Wechsler Intelligence Scales, which provide a comprehensive and psychometrically robust evaluation of cognitive ability in school-aged children and adolescents [14]. Both instruments are well-validated and commonly used in clinical and research settings to ensure reliable and comparable cognitive profiling across developmental stages. The degree of intellectual impairment was divided into profound, severe, moderate, mild, and normal.

### 2.3. Assessment of Adaptive Function

The social adaptive functioning of children with MWS was assessed using the Chinese version of Normal Development of Social Skills from Infancy to Junior High School Children scale (S-M scale). Although the S-M scale was originally developed in Japan, it has been introduced and culturally adapted for use in China and has been widely applied in Chinese clinical and research settings for the assessment of children’s adaptive/social functioning. The Chinese version of the S-M scale was standardized twice in China by a domestic collaborative group (in 1987 and 1995). These standardization efforts reported excellent test–retest reliability (r/ICC = 0.98) and high criterion validity (0.95), supporting the Chinese S-M scale as a reliable and valid measure of adaptive functioning in Chinese children [15]. In the present study, caregivers completed the questionnaire in Chinese during the follow-up visit at hospital, with assistance from trained study staff when clarification was needed; caregivers were instructed to rate the child’s typical performance rather than best performance. The scale comprises several domains relevant to daily living and social interaction. For the purposes of this study, age-appropriate items were selected based on each participant’s chronological age. Parents or primary caregivers completed the questionnaire, rating their child’s typical performance for each item. Raw scores were calculated and subsequently converted into standardized scores or developmental age equivalents according to the instrument’s manual, allowing for comparisons with typically developing peers [16,17].

### 2.4. Family Assessment Device

Family functioning was evaluated using the Family Assessment Device (FAD), a standardized self-report measure derived from the McMaster Model of Family Functioning and widely used internationally, making it applicable to families across different countries and cultural contexts. The FAD assesses key dimensions of family interactions and organization across seven domains: problem solving, communication, roles, affective responsiveness, affective involvement, behavior control, and general functioning. Items are rated on a Likert scale, and domain scores are calculated as the mean of the corresponding items, with higher scores indicating poorer family functioning. Prior work has supported the reliability and validity of the FAD and its Chinese adaptations. The Chinese General Functioning (GF) subscale demonstrated good internal consistency across multiple Chinese adolescent samples (Cronbach’s α ≈ 0.81–0.88 across samples) and satisfactory 2-week test–retest reliability (r = 0.77), with evidence of known-group validity and convergent/construct validity via expected correlations with other family functioning and psychosocial measures (convergent correlations with family-functioning measures typically in the moderate-to-high range) [18]. Standard scoring procedures were applied [19,20]. Given that psychometric support for the Chinese version is better established for the GF subscale than for other six domain subscales, the GF score was treated as the primary family functioning indicator, whereas the seven domain-level scores were analyzed in an exploratory and descriptive manner and should be interpreted cautiously. The FAD questionnaire was completed independently by the primary caregiver for each patient, in accordance with the manual’s instructions and based on the family’s typical functioning over the past two months.

### 2.5. Assessment of Behavioral Problems

The behavioral domains evaluated in this study included core autism spectrum disorder (ASD)-related behaviors, as well as co-occurring concerns regarding social interactions, emotional regulation, and sleep. ASD-related behaviors were formally assessed using the standardized Autism Behavior Checklist (ABC), a 57-item caregiver-reported instrument. Prior psychometric evaluation of the Chinese version of the ABC in a Chinese autism sample demonstrated strong reliability and validity. Interrater reliability for the five subscales and the total score was high (ICC = 0.87–0.92; Spearman’s r = 0.78–0.85), and 2-week test–retest reliability was excellent (ICC = 0.93–0.97; Spearman’s r = 0.86–0.94; all *p* < 0.01). Internal consistency was good to excellent across the five subscales and total score (Cronbach’s α = 0.75–0.96; all *p* < 0.01). Criterion validity correlations ranged from 0.39 to 0.76 (all *p* < 0.01). Confirmatory factor analysis supported the original five-factor model with acceptable fit (SRMR/”SMR” = 0.062; RMSEA = 0.052) [21].

Co-occurring symptoms were screened using a structured parent-reported questionnaire developed for this study from selected items of the Achenbach Child Behavior Checklist (CBCL). To maximize comparability across CBCL forms, we selected items that are available in most age- and sex-specific CBCL versions and that map to three prespecified domains: depressive symptoms, social impairment, and sleep problems. The exact CBCL source items (including item numbers and wording), their domain assignment, and the corresponding study questionnaire items are provided in Table S2. Each item was rated by the parent on a 3-point scale (0 = not true, 1 = sometimes true, 2 = often true) with reference to the child’s behavior during the past two months. Domain scores were calculated as the sum of item scores within each domain (depressive symptoms, social impairment, and sleep problems), with higher scores indicating greater symptom burden. No reverse-coded items were included. It is worth noting that in this study, the term “depressive symptoms” refers to a CBCL-derived symptom score based on the selected questionnaire items and does not represent a clinical diagnosis of depression [22,23]. These study-specific CBCL-derived scores were used as exploratory screening-oriented indicators rather than as fully validated independent measures of comorbid psychopathology.

### 2.6. Correlation Analysis Strategy

Correlation analyses were conducted to examine associations between family functioning and prespecified clinical/behavioral factors. The primary outcome was the general functioning score. Associations between the same factors and the other six FAD domain scores (problem solving, communication, roles, affective responsiveness, affective involvement, and behavior control) were analyzed as secondary exploratory outcomes to further characterize domain-specific association patterns.

The factors included age, paternal educational attainment, maternal educational attainment, monthly household income, degree of ID, degree of impairment in adaptive functioning, ABC total score, CBCL-derived depressive symptom score, and social impairment score, which were selected based on the study hypotheses and the published literature.

To improve transparency, we explicitly report the analytic hierarchy and the total number of correlation tests performed. A total of nine correlations were analyzed for the primary outcome (FAD general functioning), and 54 additional correlations were analyzed for the exploratory domain-level analyses (total = 63 correlation tests). No formal multiplicity correction was applied. This decision was made because the domain-level analyses were prespecified as exploratory and hypothesis-generating, and each factor–outcome pair was tested once without repeated model selection. Accordingly, findings from the exploratory domain-level analyses were interpreted cautiously, with emphasis on effect sizes (correlation coefficients) and consistency of association patterns rather than nominal statistical significance alone. Exact *p* values are reported for all analyses.

### 2.7. Statistical Analysis

The total sample consisted of 42 participants. All participants completed the family functioning questionnaire and the behavioral problems questionnaire, and there was no missing data for these measures. A cognitive assessment (IQ/DQ) was completed by 30 participants, as 12 families declined cognitive testing. Therefore, analyses involving intellectual functioning and degree of intellectual disability were conducted using complete-case data from these 30 participants. Analyses of all other variables included the full sample of 42 participants. No imputation procedures were performed.

All the statistical analyses were conducted using GraphPad software 10.1.2 (San Diego, CA, USA) or SPSS 26. Bivariate correlation analysis was conducted to examine the preliminary associations between the key continuous variables of interest. Pearson’s correlation coefficient (R) was used to assess the strength and direction of linear relationships. Subsequently, multiple linear regression analysis was employed to further investigate the predictive relationships between variables while controlling for potential covariates. Variables deemed clinically/theoretically relevant were considered for inclusion in the regression models. *p* < 0.05 was considered statistically significant.

Monthly household income and parental educational attainment were recorded as ordinal categories and coded as 1–5 for the analysis. For monthly household income, the coding was as follows: 1 ≤ CNY 5000, 2 = CNY 5000–10,000, 3 = CNY 10,000–15,000, 4 = CNY 15,000–20,000, and 5 ≥ CNY 20,000. Parental educational attainment (paternal and maternal) was similarly coded as an ordinal variable from 1 to 5 (1 = primary school to 5 = doctoral degree). In correlation and regression analyses, these variables were modeled as ordinal trend scores. Accordingly, the regression coefficient (B/β) represents the estimated change in the outcome associated with a one-category increase in income or parental education level. Because these variables represent ordered socioeconomic gradients, they were modeled as ordinal trend terms to estimate monotonic associations. The coefficients should therefore be interpreted as trend effects (per one-category increase) rather than precise interval-scaled effects.

## 3. Results

### 3.1. Cohort Analysis

A total of 42 patients with MWS were recruited for our study. The demographics of the participants are shown in Table 1. The mean age of these children was 4.90 years (median 4 years, range 2–15) and 57% of these children were female, while 43% were male. Most of them were born at term (95.2%), with a birth weight >2500 g (88.1%). Delivery mode was evenly distributed (52.4% vaginal). The primary caregiver was most commonly the mother (76.2%). The mean paternal and maternal ages were 33.12 and 30.69 years, respectively. Most fathers (73.8%) and mothers (80.9%) had a bachelor’s degree. Most families had a monthly household income of CNY 10,000–15,000 (28.6%) or 15,000–20,000 (21.4%).

### 3.2. Clinical Characteristics of the Cohort

**Figure 1:**
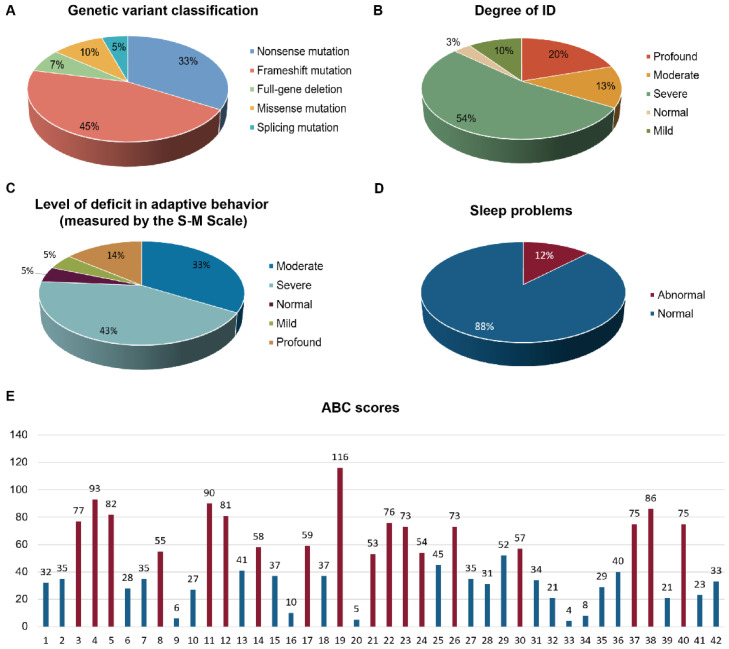
Genotype spectrum and neurodevelopmental/behavioral phenotypes of the study cohort. (**A**) Distribution of genetic variant classes. (**B**) Severity of intellectual disability (ID). (**C**) Level of adaptive behavior deficit assessed using the S-M (Social Maturity) scale. (**D**) Prevalence of sleep-related concerns based on the study-specific CBCL-derived screening items, classified as normal or abnormal. (**E**) Individual Aberrant Behavior Checklist (ABC) scores for each participant (*x*-axis); values above bars indicate the ABC score (higher scores reflect greater behavioral disturbance), red bars indicate patients with scores above the normal reference range, whereas blue bars indicate patients with scores within the normal reference range. Percentages in pie charts were calculated based on participants with available data for each measure.

The genetic variants of the patients were recorded. The most predominant variants were frameshift (45%) and nonsense mutations (33%), with smaller proportions of missense (10%), full-gene deletion (7%), and splicing mutations (5%) (Figure 1). Intellectual disability was mainly severe (≈54%), followed by profound (≈20%). Adaptive behavior deficits were most often severe (≈43%) or moderate (≈33%). Sleep-related concerns on the study-specific CBCL-derived screening items were present in approximately 12% of the participants (Figure 1).

### 3.3. Family Function of the Cohort

**Figure 2:**
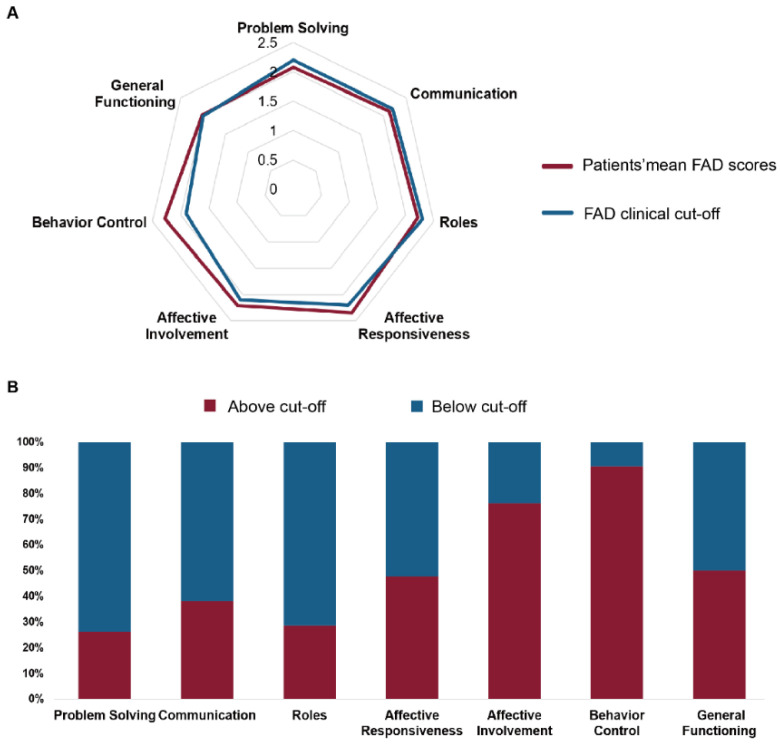
Family functioning assessed by the Family Assessment Device (FAD). (**A**) The red line denotes the mean FAD subscale scores of the patient group, whereas the blue line denotes the established FAD cut-off values for each subscale (scores above the cut-off indicate unhealthy family functioning). (**B**) Stacked bar chart showing the proportion of patients with FAD subscale scores at or above the clinical cut-off (red) and below the cut-off (blue) for each domain.

At the domain level, descriptive analyses showed that behavior control and affective involvement had the highest proportions of patients scoring above the published cut-offs, while affective responsiveness was also elevated (Figure 2). The mean subscale profiles were close to or exceeded established cut-offs in several domains, particularly affective involvement and behavior control (Figure 2A). Among the 42 patients, the number of patients exceeding the cut-off was 11 (26.2%) for problem solving, 16 (38.1%) for communication, 12 (28.6%) for roles, 20 (47.6%) for affective responsiveness, 32 (76.2%) for affective involvement, 38 (90.5%) for behavior control, and 21 (50.0%) for general functioning (Figure 2B).

### 3.4. Correlation Analyses of Potential Factors and Family Functioning

**Figure 3:**
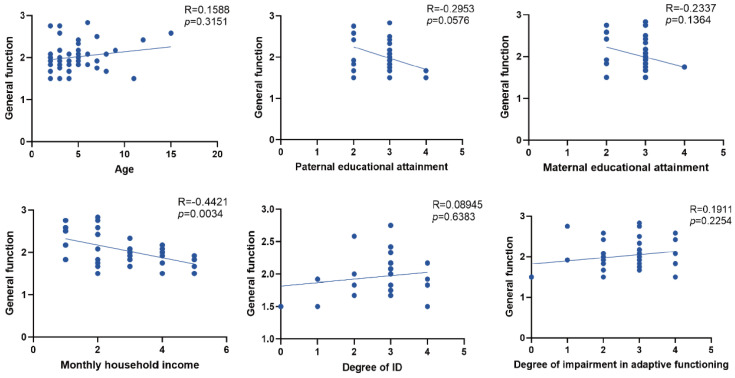
Correlates of general functioning with demographic and clinical characteristics. Correlation coefficients (R) and corresponding *p* values are shown in each panel. Higher FAD-GF scores indicate poorer overall family functioning.

We subsequently performed correlation analyses to identify potential factors associated with the patients’ family functioning. The results showed that monthly household income was significantly associated with better general family functioning (lower general functioning scores) while age, paternal education, maternal education, degree of intellectual disability, and degree of impairment in adaptive functioning were not significantly correlated with general family functioning (Figure 3). Participants who declined cognitive testing did not differ meaningfully from those included with respect to baseline characteristics (Table S3).

**Figure 4:**
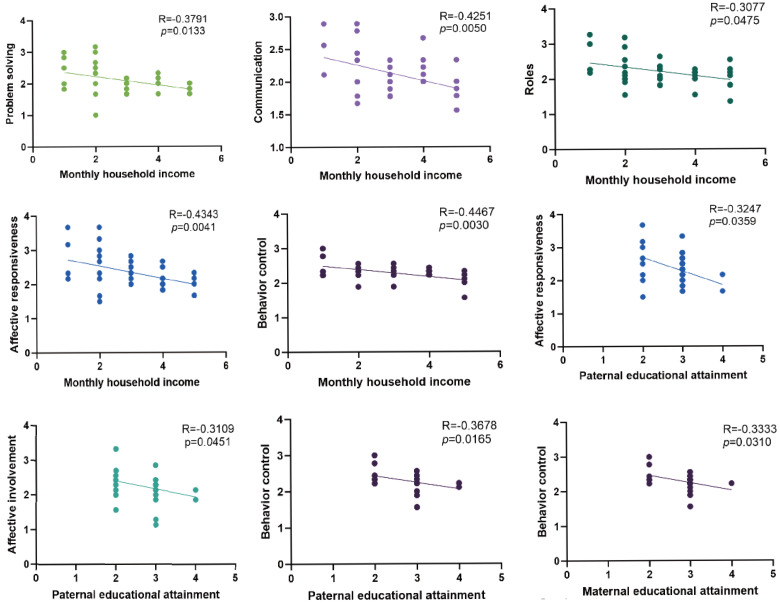
Associations between socioeconomic indicators and specific FAD domain scores. Scatterplots illustrate the relationships between FAD domain scores and monthly household income or parental educational attainment. Each dot represents one family; solid lines indicate best-fit linear regression. Correlation coefficients (R) and corresponding *p* values are provided in each panel. Higher FAD scores reflect poorer family functioning in the corresponding domain.

We also conducted exploratory analyses of the associations between demographic and clinical characteristics and domain-level FAD scores. Notably, higher household income was associated with lower scores across multiple FAD domains, including problem solving, communication, roles, affective responsiveness, and behavior control (Figure 4). Parental education was also associated with some domain scores: paternal education was correlated with affective responsiveness, affective involvement, and behavior control, while maternal education was correlated with behavior control (Figure 4). No significant associations were observed for age, severity of intellectual disability, or extent of adaptive functioning impairment across the FAD domain scores (Supplementary Figures S1–S6).

**Figure 5:**
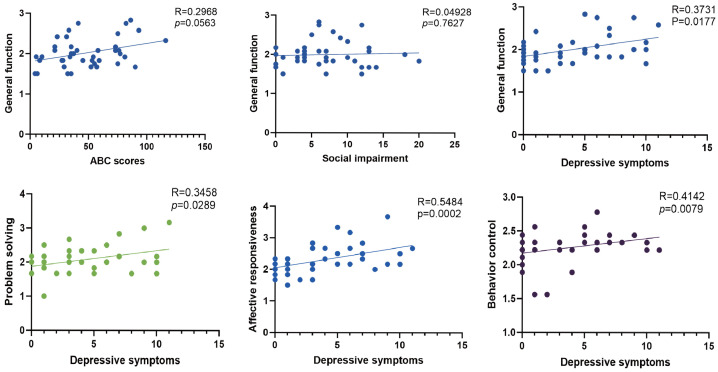
Associations between family functioning and behavioral symptoms. Each dot represents one family, solid lines indicate best-fit linear regression. Correlation coefficients (R) and corresponding *p* values are reported in each panel.

We then examined the potential correlations between family functioning and behavioral problems. Poorer general family functioning was associated with higher scores on the CBCL-derived depressive symptom domain, whereas ASD-related behavior scores showed a borderline association with family functioning, and social deficits showed no significant association with general family functioning. In addition, exploratory analyses showed that several FAD domains, including problem solving, affective responsiveness, and behavior control, were associated with higher scores on the CBCL-derived depressive symptom domain (Figure 5).

### 3.5. Multiple Regression Analysis of Predictors of Family Functioning

To identify predictors of impaired family functioning, we conducted a multiple linear regression analysis to examine whether parental educational attainment and monthly household income independently predicted general family functioning. In regression models adjusting for parental education, higher household income remained independently associated with better general functioning (standardized β = −0.443, *p* = 0.008). Paternal educational attainment and maternal educational attainment were not significant predictors. Collinearity statistics suggested acceptable levels of multicollinearity (Table 2).

## 4. Discussion

To our knowledge, this study provides the first systematic characterization of family functioning in families of children with MWS and demonstrates a pattern of pervasive impairment. Among the variables examined, household income emerged as a specific factor independently associated with general family functioning, with higher income linked to better functioning. This finding underscores the importance of considering socioeconomic context when interpreting family functioning in this population.

Families of children with MWS appeared to experience difficulties in family functioning, with mean FAD scores in several domains approaching or exceeding cut-off values. At the descriptive level, the highest proportions of scores above the cut-off were observed in behavior control and affective involvement, while affective responsiveness was also elevated in a substantial proportion of families. However, because psychometric support in Chinese populations is more robust for the GF subscale than for the other FAD subscales, these domain-level patterns should be viewed as exploratory descriptive signals rather than firm evidence of specific dysfunction profiles. Previous studies showed that families of children with cognitive impairments exhibit poorer family functioning than those of typically developing children. Evidence further indicates that family functioning is more problematic in families of children with ASD or ADHD than in families of neurotypical peers [4,24,25]. These findings suggest that difficulties in family functioning may reflect a broad challenge faced by families caring for children with complex developmental and chronic health conditions.

In the present study, monthly household income emerged as the most salient predictor of overall family functioning. While parental educational attainment also showed correlations with certain domains, household income remained the most robust socio-economic correlate of overall family functioning in our model, aligning with prior evidence from multiple linear regression analyses showing that greater socio-ecological resilience among parents is associated with reduced parenting stress, which, in turn, may contribute to more adaptive family functioning [11,26]. Families affected by rare diseases often shoulder substantial financial strain, arising from both direct costs (medical care, rehabilitation therapies, and special education) and indirect costs (reduced household income when a parent leaves the workforce or cuts back on employment to provide intensive caregiving). In this context, higher household income may reflect greater access to material, caregiving, and rehabilitative resources, which might help reduce chronic family stress. Conversely, limited financial resources may be associated with greater caregiver burden and strain within the family.

Prior studies suggest that greater disease severity may be associated with poorer family functioning [4,5,6]. However, in our sample, neither the level of intellectual disability nor the extent of adaptive impairment was significantly related to family functioning. This null finding may reflect limited statistical power due to the relatively small sample size, as well as the restricted set of indicators used to index disease severity. Future studies should incorporate a larger cohort and a broader, multidimensional assessment of clinical severity to more robustly evaluate its association with family functioning.

Our findings further suggest that poorer family functioning may be associated with higher levels of behavioral and emotional difficulties, including ASD-related behavioral problems and higher scores on the CBCL-derived depressive symptom. This pattern appears broadly consistent with previous studies reporting associations between family functioning and problem behaviors in children with intellectual and developmental disabilities [3,27]. However, these findings should be interpreted cautiously. The present cross-sectional data do not permit conclusions about causality or directionality. It is also possible that greater child behavioral and emotional difficulties place additional strain on family functioning, rather than family functioning acting as a primary driver of these outcomes. Accordingly, these results are best viewed as preliminary and hypothesis-generating. Future longitudinal studies, ideally using fully validated symptom measures, are needed to clarify the temporal and potentially bidirectional relationships between family functioning and child behavioral outcomes.

Overall, our findings suggest that socioeconomic context, particularly household income, should be explicitly considered when evaluating family functioning in families of children with MWS. Future research should examine whether this association can be replicated in larger samples and clarify the mechanisms through which financial context may relate to family adaptation over time.

Several limitations should be noted. First, this study was based on a single-center convenience sample recruited through investigator-initiated online contact. Therefore, selection bias cannot be excluded, and the generalizability of the findings is limited. Second, because of the cross-sectional design, the observed relationships should be interpreted as associations only, and no causal or determinant-style inferences can be made. Third, co-occurring emotional, social, and sleep-related concerns were assessed using study-specific scores derived from selected CBCL items rather than fully validated standalone measures of comorbid psychopathology. These variables should therefore be regarded as exploratory, screening-oriented indicators and interpreted with caution. Fourth, although the FAD General Functioning subscale served as the primary family-functioning indicator in this study, the other six FAD domains were examined at an exploratory level only. The psychometric support for the Chinese FAD is more established for the General Functioning subscale than for detailed interpretation of all seven domains. Finally, the domain-level analyses were exploratory and no formal multiplicity correction was applied; therefore, these findings should be considered hypothesis-generating and interpreted cautiously because of the risk of type I error.

## 5. Conclusions

In summary, our findings indicate that families of children with MWS commonly experience substantial functional difficulties, and higher household income was independently associated with better general functioning. These results underscore household income as a specific socioeconomic factor associated with family functioning in this population. Future studies are needed to replicate this association in larger samples and to clarify how socioeconomic context may shape family adaptation over time.

## Figures and Tables

**Table 1:** Demographics of the participants in this study.

Variable	Characteristic	Number of Participants (*n* = 42)	Percent
Gender	Male	18	43%
	Female	24	57%
Mean age (years)	4.90		
Median age and range (min–max, years)	4 (2–15)		
Preterm delivery	Yes	2	4.8%
	No	40	95.2%
Birth weight (g)	<1500	1	2.4%
	1500–2500	4	9.5%
	>2500	37	88.1%
Mode of delivery	Cesarean section	20	47.6%
	Vaginal delivery	22	52.4%
Birth asphyxia	Yes	3	7.1%
	No	39	92.9%
Feeding method after birth	Breastfeeding	10	23.8%
	Formula feeding	11	26.2%
	Mixed feeding	21	50%
Mean age of father		33.12	
Mean age of mother		30.69	
Paternal educational attainment	Primary school	0	0%
	Middle school	9	21.4%
	Bachelor’s degree	31	73.8%
	Master’s degree	2	4.8%
	Doctoral degree	0	0%
Maternal educational attainment	Primary school	0	0%
	Middle school	7	16.7%
	Bachelor’s degree	34	80.9%
	Master’s degree	1	2.4%
	Doctoral degree	0	0%
Primary caregiver	Father	3	7.1%
	Mother	32	76.2%
	grandparents	6	14.3%
	Babysitter	1	2.4%
Monthly household income	<CNY 5000	5	11.9%
	CNY 5000–10,000	10	23.8%
	CNY 10,000–15,000	12	28.6%
	CNY 15,000–20,000	9	21.4%
	>CNY 20,000	6	14.3%

max: maximum, min: minimum.

**Table 2:** Multiple linear regression analysis predicting general functioning.

Predictor	B	SE	β	t	*p*
Paternal educational attainment	−0.121	0.129	−0.169	−0.943	0.352
Maternal educational attainment	0.006	0.146	0.008	0.043	0.966
Monthly household income	−0.126	0.045	−0.443	−2.782	0.008

B, unstandardized regression coefficient; SE, standard error; β, standardized coefficient.

## Data Availability

The original contributions of this study are provided in the article and its Supplementary Materials. The data presented in this study are available on request from the corresponding author. The data are not publicly available due to privacy restrictions.

## Supplementary Materials

The following supporting information can be downloaded at https://www.mdpi.com/article/10.3390/healthcare14070931/s1, Table S1: Genotype spectrum and clinical characteristic of the study cohort; Table S2: Co-occurring symptoms questionnaire; Table S3: Baseline Characteristics of Participants by Completion of the Intelligence Test; Table S4: Missing data summary (N available for each measure); Figure S1: Scatter plots showing correlations between problem-solving scores and age, paternal and maternal educational attainment, degree of intellectual disability, degree of impairment in adaptive functioning, ABC scores, and social impairment; Figure S2: Scatter plots showing correlations between communication scores and age, paternal and maternal educational attainment, degree of intellectual disability, degree of impairment in adaptive functioning, ABC scores, social impairment, and depression; Figure S3: Scatter plots showing correlations between roles scores and age, paternal and maternal educational attainment, degree of intellectual disability, degree of impairment in adaptive functioning, ABC scores, social impairment, and depression; Figure S4: Scatter plots showing correlations between affective responsiveness scores and age, maternal educational attainment, degree of intellectual disability, degree of impairment in adaptive functioning, ABC scores, and social impairment.; Figure S5: Scatter plots showing correlations between affective involvement scores and age, maternal educational attainment, monthly household income, social impairment, degree of impairment in adaptive functioning, ABC scores, degree of intellectual disability, and depression; Figure S6: Scatter plots showing correlations between behavior control scores and age, degree of intellectual disability, degree of impairment in adaptive functioning, ABC scores, and social impairment.
