# Supplementary material for: Profiles and Predictors of Family Functioning in Families of Children with Mowat–Wilson Syndrome: A Cross-Sectional Survey
Source: Healthcare (Basel). 2026 Apr 2;14(7):931. doi: 10.3390/healthcare14070931 (PMC13073676; doi:10.3390/healthcare14070931)
Supplement: Supplementary file 1 [file healthcare-14-00931-s001.zip › healthcare-4121543-supplementary.pdf]

Supplementary Table S1. Genotype spectrum and clinical characteristic of the study cohort

Variable	Characteristics	Number of Participants	Percent
Genetic variant classification	Nonsense mutation	14	33%
	Frameshift Mutation	19	45%
	Full-gene deletion	3	7%
	Missense mutation	4	10%
	Splicing mutation	2	5%
Degree of ID	Normal	1	3%
	Mild	3	10%
	Moderate	4	13%
	Severe	16	54%
	Profound	6	20%
Degree of impairment in adaptive functioning	Normal	2	5%
	Mild	2	5%
	Moderate	14	33%
	Severe	18	43%
	Profound	6	14%
Sleep problems	Yes	5	12%
	No	37	88%

ID intellectual disability

Supplementary Table S2. Co-occurring symptoms questionnaire

CBCL 4–16 (parent report)
**Sleep Problem**
• Nightmares.<br>• Sleeps less than most children.<br>• Sleeps more than most children (not just staying in bed).<br>• Sleep-talking or sleep-walking.<br>• General sleep difficulties (describe if needed).
**Social withdrawal**
• Does not get along with other children / avoids peer interaction.<br>• Prefers to be alone.<br>• Not liked by other children.<br>• Refuses to talk / is silent.<br>• Keeps things to self; does not share concerns.<br>• Withdrawn / socially isolated.
**Depressive symptoms**
• Often says they feel lonely.<br>• Cries a lot.<br>• Feels or complains that nobody likes them.<br>• Feels worthless or has low self-esteem.<br>• Feels excessively guilty.<br>• Seems unhappy, sad, or depressed.<br>• Complains/whines frequently.<br>• Worries a lot.
**CBCL 2–3 (parent report)**
**Sleep Problem**
• Cannot sleep alone.<br>• Has trouble falling asleep.<br>• Nightmares.<br>• Refuses to go to bed at night.<br>• Sleeps less than most children (day and night).<br>• Talks or screams loudly during sleep.<br>• Wakes up often during the night.
**Social withdrawal**
• When spoken to, does not want to answer questions.<br>• Does not want to leave home.<br>• Seems emotionally cold toward people.<br>• Shows little interest in surrounding things.

• Poor social engagement / avoids interacting with others.
**Depressive symptoms**
• Easily discouraged / easily loses heart.<br>• Unhappy for no clear reason.<br>• Unhappy, sad, or depressed.<br>• Tends to worry / be troubled.

Selected from items of the Achenbach Child Behavior Checklist
Response options (0/1/2):
0 = not present
1 = mild / sometimes
2 = clear / often

Supplementary Table S3. Baseline Characteristics of Participants by Completion of the Intelligence Test

	**Participants who completed the intelligence test**	**Participants who did not complete the intelligence test**	**P**
Age	5.00（3.00，6.25）	3.00（2.00,6.00）	0.108
Birth weight	3330（2887,3725）	3205（3000,3757）	0.794
Boy, n (%)	13 (43.3)	5 (41.7)	0.600
Preterm delivery, n (%)	2 (6.7)	2 (16.7)	0.678
Cesarean section	14 (46.7)	6 (50)	0.845

Supplementary Table S4. Missing data summary (N available for each measure)

**Measure / Variable**	**Total sample (N)**	**N available**	**N missing**	**% missing**	**Missing-data handling / Analysis sample**
Family functioning questionnaire	42	42	0	0.0%	Full sample used (no missing data)
Behavioral problems questionnaire	42	42	0	0.0%	Full sample used (no missing data)

Cognitive assessment: IQ/DQ	42	30	12	28.6%	Complete-case analysis (N=30)

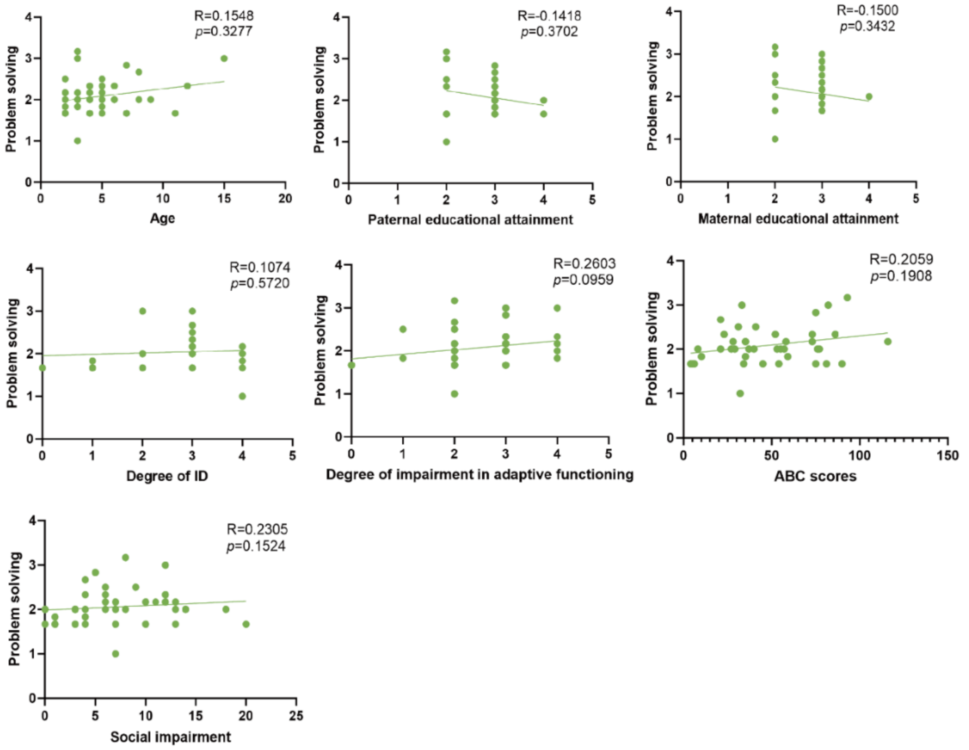

Supplementary Figure S1. Scatter plots showing correlations between problem-solving scores and age, paternal and maternal educational attainment, degree of intellectual disability, degree of impairment in adaptive functioning, ABC scores, and social impairment.

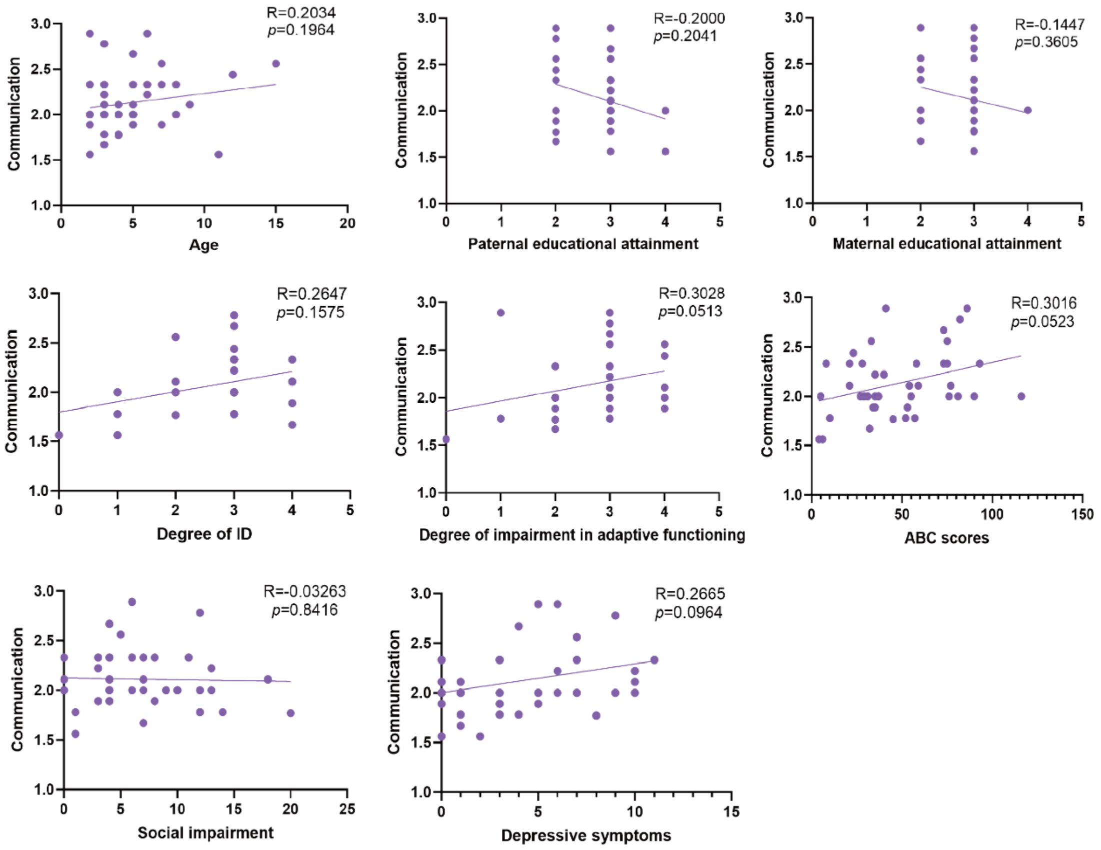

Supplementary Figure S2. Scatter plots showing correlations between communication scores and age, paternal and maternal educational attainment, degree of intellectual disability, degree of impairment in adaptive functioning, ABC scores, social impairment, and depression.

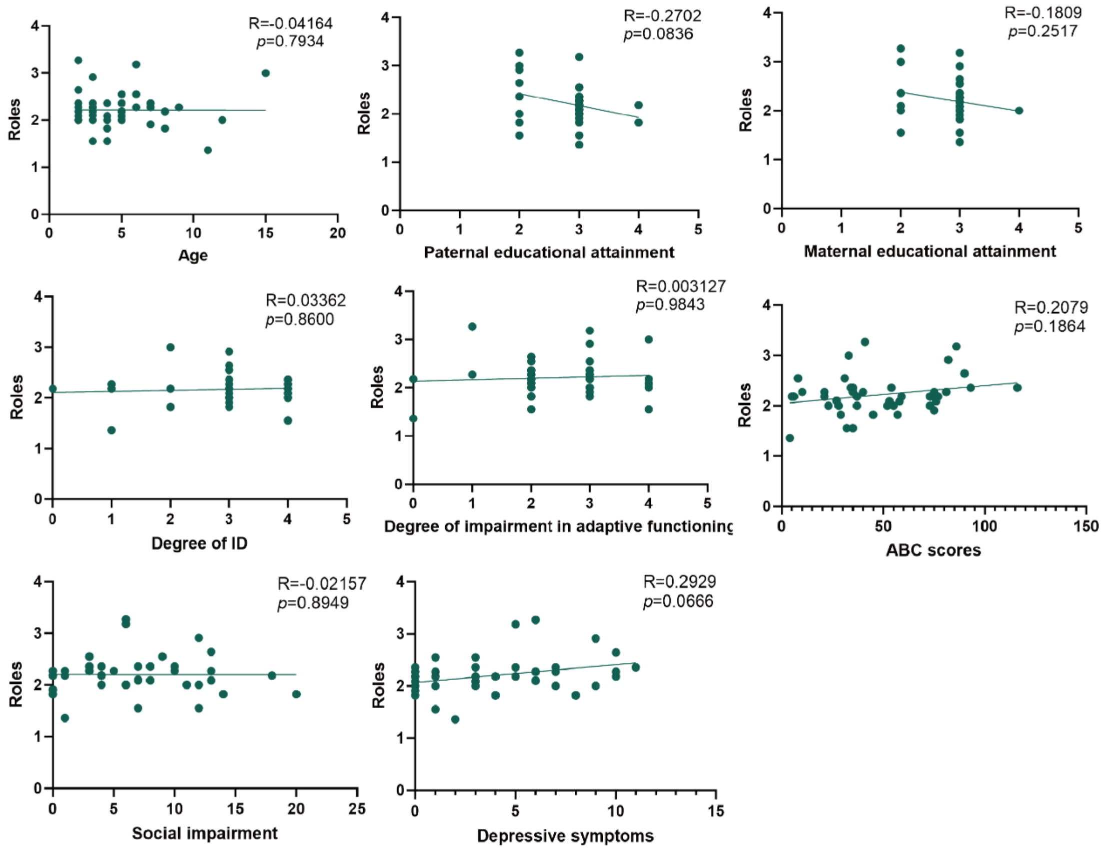

Supplementary Figure S3. Scatter plots showing correlations between roles scores and age, paternal and maternal educational attainment, degree of intellectual disability, degree of impairment in adaptive functioning, ABC scores, social impairment, and depression

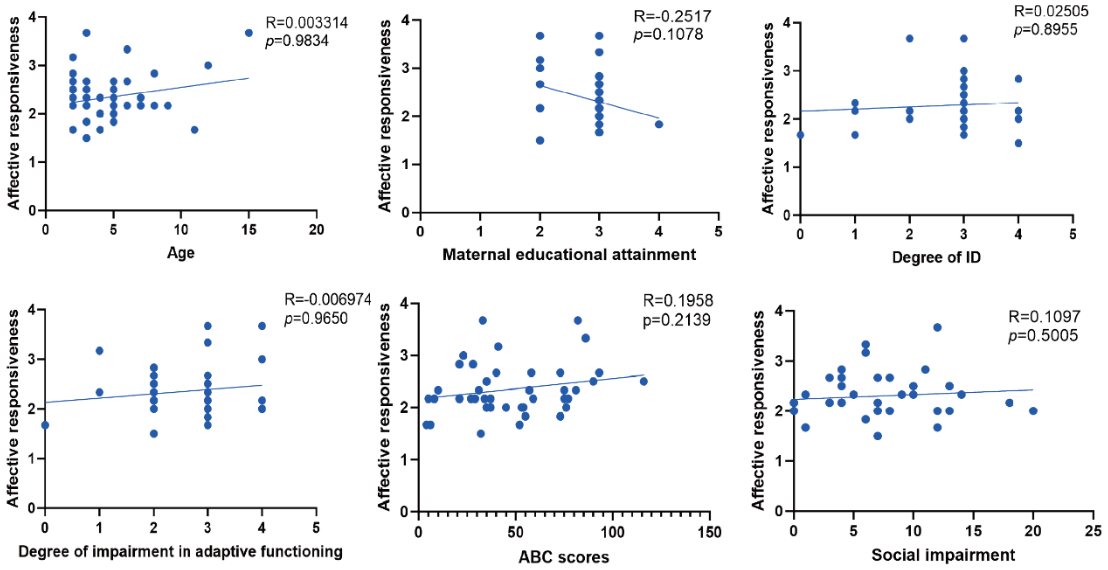

Supplementary Figure S4. Scatter plots showing correlations between affective responsiveness scores and age, maternal educational attainment, degree of intellectual disability, degree of impairment in adaptive functioning, ABC scores, and social impairment.

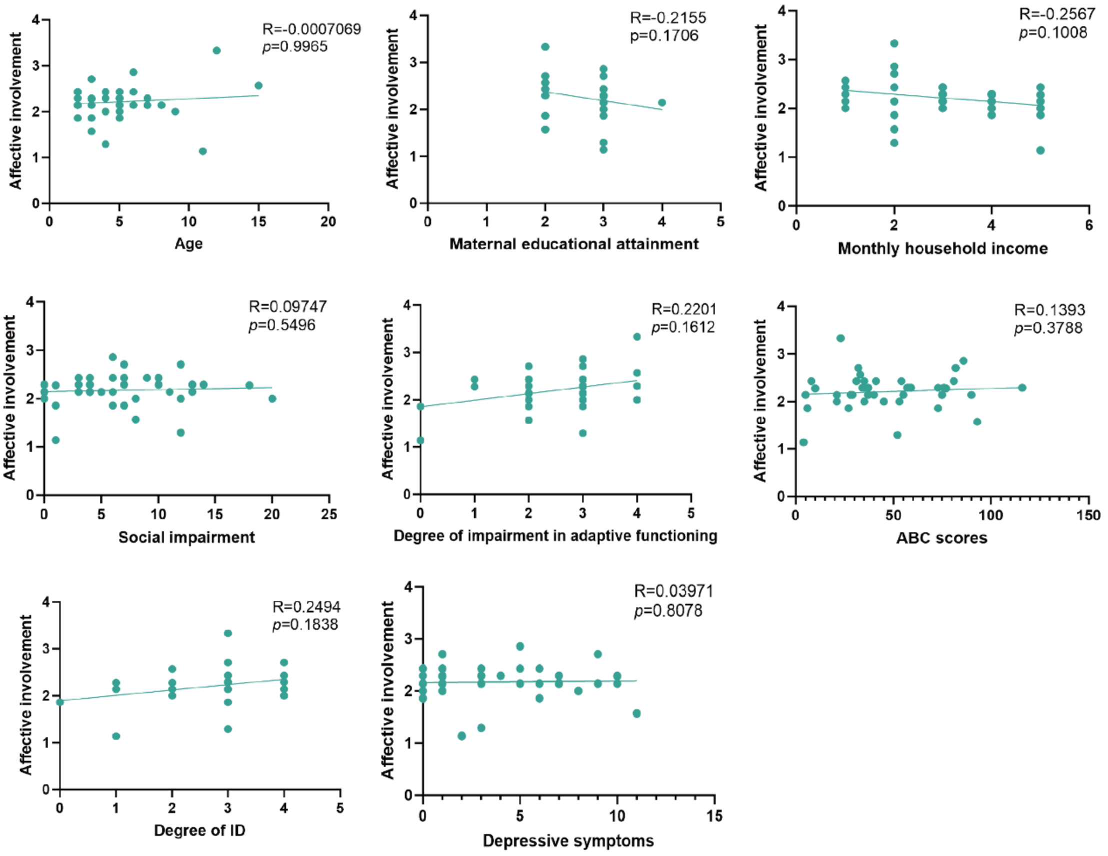

Supplementary Figure S5. Scatter plots showing correlations between affective involvement scores and age, maternal educational attainment, monthly household income, social impairment, degree of impairment in adaptive functioning, ABC scores, degree of intellectual disability, and depression.

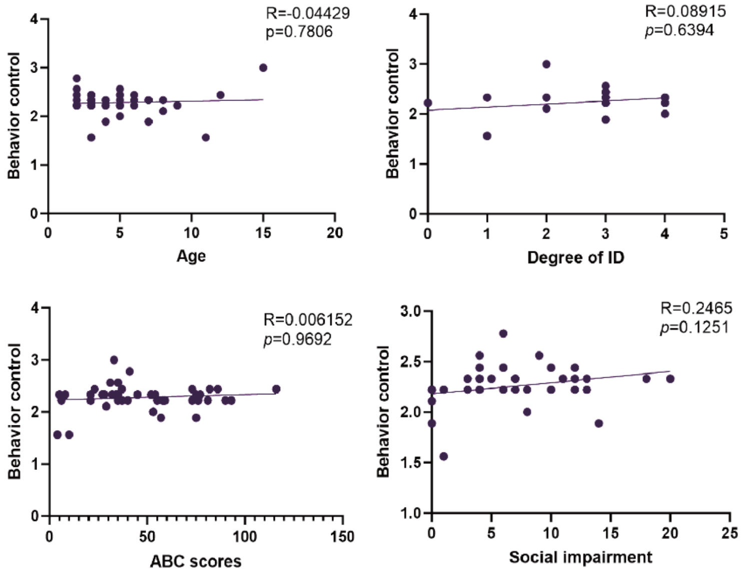

Supplementary Figure S6. Scatter plots showing correlations between behavior control scores and age, degree of intellectual disability, degree of impairment in adaptive functioning, ABC scores, and social impairment.
